# High prevalence of selected viruses and parasites and their predictors in Malawian children

**DOI:** 10.1017/S0950268819000025

**Published:** 2019-02-22

**Authors:** Y.-M. Fan, S. Oikarinen, K.-M. Lehto, N. Nurminen, R. Juuti, C. Mangani, K. Maleta, H. Hyöty, P. Ashorn

**Affiliations:** 1 Center for Child Health Research, Tampere University, Faculty of Medicine and Health Technology and Tampere University Hospital, Tampere, Finland; 2 Department of Virology, Faculty of Medicine and Health Technology, Tampere University, Tampere, Finland; 3 EPID Research Oy, Espoo, Finland; 4 College of Medicine, University of Malawi, Blantyre, Malawi; 5 Department of Paediatrics, Tampere University Hospital, Tampere, Finland

**Keywords:** Infection, parasite, predictor, prevalence, virus

## Abstract

Enteric pathogens have been related to child undernutrition. Whereas there are lots of data on enteric bacterial microbiota and infections, much less is known about the incidence of prevalence of intestinal colonisation with viruses or important parasitic species. This study assessed the presence of selected viruses and parasites in stools of 469, 354, 468 Malawian children at 6, 12 and 18 months. We also assessed environmental predictors of the presence of viruses and parasites among 6-month infants. Microbial presence was documented using real-time polymerase chain reaction (PCR). Enteroviruses were identified in 68%, 80% and 81% of the stool samples at 6, 12 and 18 months children, rhinovirus in 28%, 18% and 31%, norovirus in 24%, 22% and 16%, parechovirus in 23%, 17% and 17%, rotavirus in 3%, 1% and 0.6%, *Giardia lamblia* in 9.6%, 23.5% and 26%, and *Cryptosporidium* (spp.) in 6%, 8% and 2% of the 6, 12 and 18 months stool samples. Dry season (May–October) was associated with a low infection rate of enterovirus, norovirus and *Cryptosporidium* (spp.). Higher father's education level, less number of person in the household and higher sanitation were associated with a low infection rate of enterovirus, norovirus and rotavirus, respectively. The results suggest that the prevalence of asymptomatic viral and parasitic infections is high among Malawian children and that the family's living conditions and seasonality influence the rate of infections.

## Introduction

Stunting, defined as children's length-for-age or height-for-age, is more than two standard deviations below the World Health Organisation (WHO) Child Growth Standard median. Globally, it is estimated that 165 million children younger than 5 years were affected by stunting in 2011. The prevalence has been decreasing during the past decades, but is still high in Sub-Saharan Africa and South Asia (36% and 27%) [1]. In childhood, stunting is associated with an increased risk of morbidity and mortality as well as delayed mental and motor development [1]. Impaired early childhood undernutrition in turn has been associated with lower educational achievement, lower adult productivity and earning potential [2].

The most common cause of stunting is believed to be results of inadequate dietary intake, frequent infections or combination of both. In recent years, environmental enteropathy, also known as environmental enteric dysfunction (EED), has been studied as another possible cause [3, 4]. This condition is characterised by intestinal inflammation, diffuse villous atrophy and impaired nutrient absorption and growth. It is believed to have an infectious origin [5], but neither antibiotic nor probiotic treatment seems to modify its course [6, 7]. Viral and parasitic infections would not be affected by antibacterial interventions but they do cause intestinal inflammation response [8, 9]. Furthermore, whole-transcriptome analysis from Malawian children with varying states of EED showed that 57% transcripts with increased expression in EED were reported to respond to viral infection [10]. Viral or parasitic infections of the gastrointestinal tract are also often common but asymptomatic [11–18]; for instance, in a pilot study of 44 6-month-old infants in rural Malawi, we detected enteroviral RNA in 50% of the tested stool samples [19]. The stool samples were collected as part of the same intervention trial as the current study. Given all these findings, it seems plausible that asymptomatic intestinal viral or parasitic infections would be contributing to EED.

To get a more comprehensive picture of the frequency of intestinal viral or parasitic infections among children in a low-income setting, we determined the prevalence of five viruses- enterovirus, norovirus, rhinovirus, parechovirus, rotavirus and two parasites – *Giardia lamblia* and *Cryptosporidium* species in stools from 6, 12 and 18 months Malawian children. We focused on these pathogens because they are widely spread and reflect the hygienic conditions where the children are living. As an attempt to identify potential targets for preventive interventions, we also sought to identify major environmental or socio-demographic predictors for being infected with selected viruses or parasites among 6-month infants.

## Methods

### Study design, study subjects and ethics

This study was a cross-sectional study using data and stool samples that were collected from rural Malawi. The 6-month infants were recruited to a clinical dietary supplementation trial between January 2008 and May 2009, at that point we examined the infants, interviewed their guardians and collected and stored a stool sample from them. The infants were followed up for 12 months and stool samples were also collected when they were 12- and 18-month-old.

**Fig. 1.**
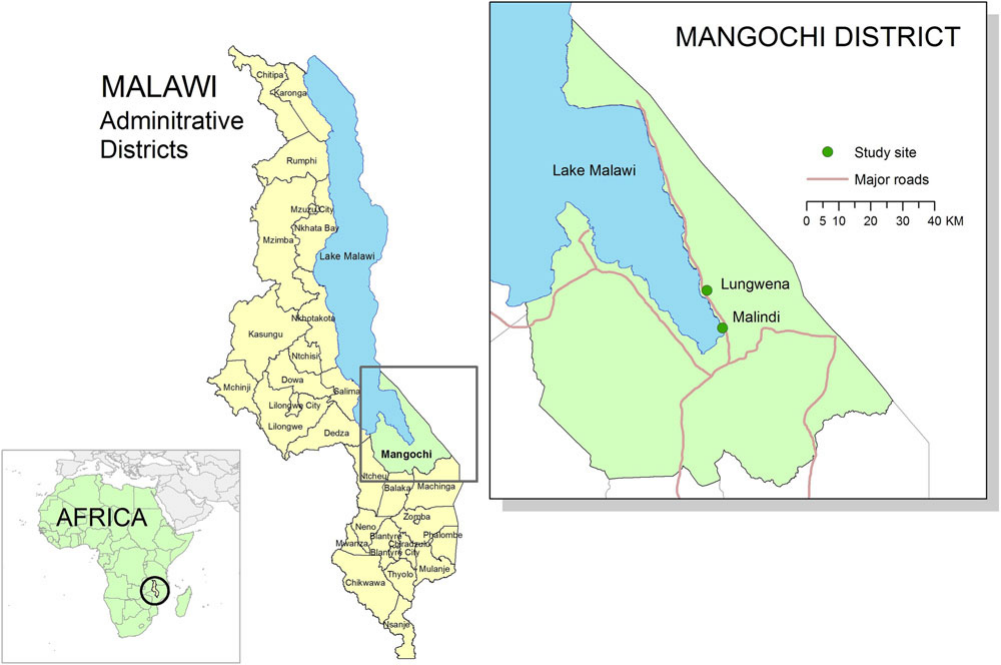
Map of study sites (Lungwena and Malindi). Source: Data layer for Africa map downloaded from http://www.thematicmapping.org, 2015 available under a Creative Commons Attribution-Share Alike License 3.0 (borders may not be completely accurate). All other data layers downloaded from Malawi Spatial Data Portal, 2015 (http://www.masdap.mw).

Details of this trial, designated Lungwena Child Nutrition Intervention Study (LCNI-5), have been described elsewhere [20]. In brief, the study population comprised 840 6-month-old infants, who were enrolled to a clinical trial assessing the effect of dietary supplementation with lipid-based nutrient supplements for 12 months on early childhood growth (registration ID: NCT00524446, at https://clinicaltrials.gov/). This trial was conducted in Lungwena and Malindi, two rural Malawian communities. Malindi is about 17 km from Mangochi town and had on average better access to electricity, clean water, sanitation and missionary hospital and a more educated population than Lungwena, a more rural site about 32 km from the town. Both areas are along the shores of Lake Malawi, Malawi. (Fig. 1).

The trial adhered to the principles of the Declaration of Helsinki and regulatory guidelines in Malawi. Written informed consent was obtained from the participants’ guardians and the trial protocol was reviewed and approved by the College of Medicine research and ethics committee (University of Malawi) and the ethical committee of the Pirkanmaa Hospital District, Finland.

### Specimen collection, data collection and storage

All stool samples were collected within 14 days after the enrolment. The guardians of the trial participants were given a container to collect the stool samples from the participants, and they brought the sample to the health centre afterwards. We collected data with picture calendar recorded by caretaker (guardian). Throughout the study, the guardians were asked to record on a daily basis the presence or absence of illness symptoms in picture calendars that were provided every 2 weeks. The calendar had separate rows for different days up to 2 weeks and separate columns for the following symptoms: (1) fever; (2) cough; (3) diarrhoea (⩾3 stools/d); and (4) other. The first 3 symptom columns had pictures describing them to assist the guardians in identifying the correct area to record the information. The guardian reports on the calendars did not include the recording of temperature for fever or the number of stools for diarrhoea. The recorded information was reviewed and cross-checked by fieldworkers at each 2 weekly food-delivery visit for completeness. Those participants carrying the pathogens but did not have illness symptoms and looked ‘healthy’ from their caretakers’ views comprised our study subjects. From the information we collected at enrolment, 31 children had diarrhoea in the last 14 days, 22 during the last 7 days and 11 in the last 2 days. The pathogen infections were identified by PCR from stool samples collected at 6, 12 and 18 months.

Time between defecation and stool freezing was typically 1–3 h, maximally 6 h. About 1 g stool sample was aliquoted by a laboratory technician to a screw cap collection tube. The samples were stored for a maximum of 1 week at −20 °C in the study site, 2–4 years at −40 °C at a central laboratory in Mangochi and 2–7 months at −80 °C at the University of Tampere where the analysis was done. The sample shipment from Malawi to Tampere took place in dry ice.

### Sample preparations, nucleic acid extraction and real-time PCR

Frozen stool samples were thawed and processed into 10% (w/v) suspension in HANKS medium containing 0.2% BSA. Nucleic acids were isolated using QIAamp Viral RNA Mini Kit (Qiagen, Hilden, Germany) from the 10% faecal suspension and real-time PCR was used to detect enteroviruses, rhinoviruses, noroviruses, parechoviruses and rotaviruses [21, 22] as well as *Giardia lamblia* and *Cryptosporidium* (spp.) parasites as previously reported [23]. The primers and probes used for PCR were shown in supplementary Table S1. For each pathogen, we ran three times PCR and positive was identified if PCR were positive at two or three times.

### Collection of background information and laboratory tests

The data on participants’ socioeconomic background were collected through personal interviews during enrolment. At enrolment, the participants’ blood haemoglobin concentration was also measured from a venous sample using cuvettes and a reader (HemoCue AB, Angelholm, Sweden). Malaria was diagnosed microscopically from Giemsa stained thick and thin blood films. Each smear was read by two independent microscopists and discordant results were re-read by a third microscopist.

### Anthropometric measurements

Anthropometric measurements were carried out by three trained research assistants during the enrolment visit. Unclothed infants were weighed using an electronic infant weighing scale (SECA 735; Chasmors Ltd, London, England) and weights were recorded to the nearest 10 g. The length was measured to the nearest 1 mm using a high-quality length board (Kiddimetre; Raven Equipment Ltd, Essex, England). Anthropometric indices (weight-for-age *Z* score = WAZ, length-for-age *Z* score = LAZ and weight-for-length *Z* score = WLZ) were calculated using WHO Child Growth Standards (2010 STATA igrowup package) [24]. Age for all participants was verified before enrolment objectively by crosschecking documented birth dates in health passports and under-five cards.

### Statistical analysis

Proportions, means and standard deviations (s.d.) were compared between groups by using Student's *t* test for continuous variables and Fisher's exact test for proportions. Frequencies were used to determine the prevalence of viruses and parasites. Bivariate analysis was used to determine the association between potential predictors including age, sex, growth indexes, age of the main guardian, number of persons and children <5 years of age in household, parents’ education level, socioeconomic status, number of animals in the house, anaemia, malaria, source of drinking water, household sanitation (latrine), site (Lungwena or Malindi), seasonality (dry or rainy season when stool samples were collected) and presence of viruses and parasites. Those predictors that were found to be at the *P* < 0.1 level were included in the multivariate logistic regression model. Age, sex, socioeconomic status and number of animals in household were forced into the model since they have been found to be significant in the literature and are potential confounders [25–27]. Odds ratios with 95% CI were used to measure the association between predictors and presence of viruses and parasites at statistical significance of *P* < 0.05. Statistical analyses were performed using Stata 13.1 (Stata Corp, College Station, TX, USA).

## Results

### Characteristics of study subjects

**Fig. 2.**
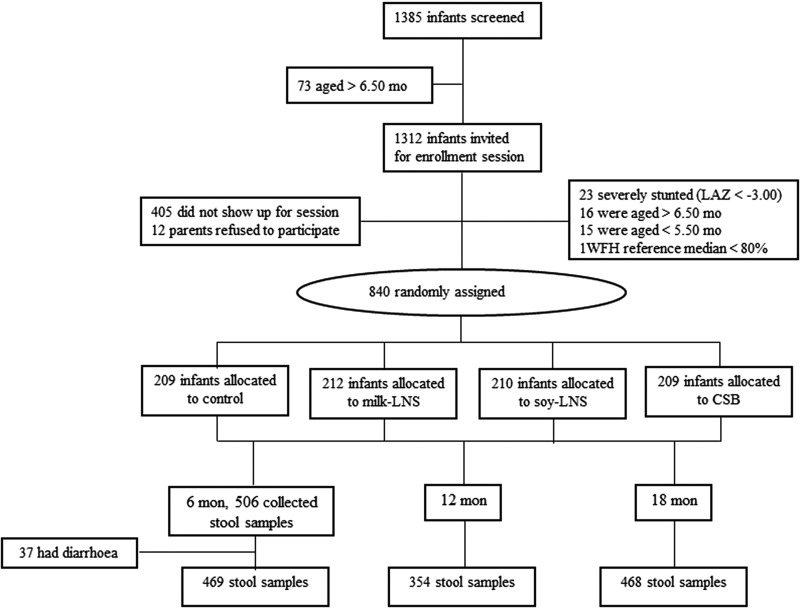
Flow diagram of participant progress throughout the study. CSB, corn-soy blend; LAZ, length-for-age Z-scores; milk-LNS, lipid-based nutrient supplement with milk protein base; soy-LNS, lipid-based nutrient supplement with soy protein base; WFL, weight-for-length.

Of the 840 LCNI-5 trial participants, we had 506 stored stool samples from 6-month infants. Among them, 37 were found to have diarrhoea at sample collection day and were excluded from this study. There were 11 infants who had diarrhoeal during the past 2 days when the samples were collected. However, these participants were included in the study subjects. Stool samples of 354 and 468 children were available at age of 12 and 18 months, respectively (Fig. 2). At 6 months of age, the mean LAZ, WAZ and WLZ of these participants were −1.65, −0.79 and +0.45, respectively. Approximately 13% of them had malaria parasitaemia in their blood. Infants who participated in the main intervention trial but were not included in this sub-study were on average similar to the included ones, except that they were slightly older and less often enrolled from Lungwena health centre (Table 1).

**Table 1.** Characteristics of participants

	Study participants (*N* = 469)	Subjects without stool samples or with diarrhoea (*N* = 371)	*P* value
Proportion of boys	47.3%	53.1%	0.110
Mean (s.d.) infant age, months	5.98 (0.25)	6.09 (0.22)	<0.001
Mean (s.d.) weight-for-age *z*-score (WAZ)	−0.79 (1.10)	−0.79 (1.13)	0.499
Mean (s.d.) length-for-age *z*-score (LAZ)	−1.65 (1.00)	−1.66 (1.03)	0.4403
Mean (s.d.) weight-for-length *z*-score (WLZ)	0.45 (1.02)	0.45 (1.08)	0.473
Mean (s.d.) age of the main guardian, years	28 (9)	28 (10)	0.342
Mean (s.d.) number of persons in household	4.8 (1.8)	4.9 (1.8)	0.196
Mean (s.d.) number of children <5 years of age in household	1.6 (0.7)	1.6 (0.6)	0.145
Mean (s.d.) mothers’ education, completed years	3.5 (3.4)	3.6 (3.2)	0.328
Mean (s.d.) fathers’ education, completed years	4.1 (3.8)	4.3 (4.0)	0.269
Mean (s.d.) number of economic support person in the family	1.5 (1.1)	1.7 (1.3)	0.076
Mean (s.d.) number of animals in the house	5.9 (10.6)	5.2 (8.0)	0.166
Proportion with moderate anaemia (Hb <80 g/l)	15.0%	14.9%	1.00
Proportion with positive malaria microscopy	13.0%	14.6%	0.539
Proportion with unsafe source of drinking water^a^	10.2%	6.2%	0.056
Proportion with household latrine	92.8%	93.8%	0.673
Proportion enrolled from Lungwena site	62.9%	48.5%	<0.001
Proportion of sample collection at rainy season, November–April	58.4%	53.6%	0.183

s.d., standard deviation.

a Source of drinking water from unprotected well, lake or pond.

*P*-value obtained from Student's *t* test (continuous variables) or Fisher's exact test (proportions).

### Prevalence of viruses and parasites in stool samples

A total of 320 (68.2%), 284 (80.2%) and 380 (81.2%) of the stool samples contained enteroviral RNA at 6, 12 and 18 months, respectively. Rhinovirus was detected in 130 (27.7%), 65 (18.4%) and 146 (31.2%) samples; norovirus in 110 (23.5%), 79 (22.3%) and 74 (15.8%); parechovirus in 106 (22.6%), 60 (17%) and 77 (16.5%); and rotavirus in 15 (3.2%), 4 (1.1%) and 3 (0.6%) samples from 6, 12 and 18 months, respectively (Table 2). A total of 45 (9.6%), 83 (23.5%) and 123 (26.3%) samples were positive for *Giardia lamblia* and 30 (6.4%), 28 (7.9%) and 9 (1.9%) for *Cryptosporidium* (spp.) from 6, 12 and 18 months, respectively (Table 2). At least one virus or parasite was detected in 414 (88.3%), 317 (89.5%) and 418 (89.3%) stool samples at 6, 12 and 18 months. Two or more microbes were detected in 234 (49.9%), 188 (53.1%) and 262 (56%) and three or more microbes in 86 (18.3%), 72 (20.7%) and 105 (22.4%) of 6, 12 and 18 months samples.

**Table 2.** Prevalence of viruses and parasites in 6, 12 and 18-month-old Malawian children

Virus/Parasite	Age	*N*	*N* of positive	% of positive	95% CI
Enterovirus	6-month	469	320	68.2	64.0–72.5
	12-month	354	284	80.2	76.1–84.4
	18-month	468	380	81.2	77.6–84.7
Rhinovirus	6-month	469	130	27.7	23.7–31.8
	12-month	354	65	18.4	14.3–22.4
	18-month	468	146	31.2	27.0–35.4
Norovirus	6-month	469	110	23.5	19.6–27.3
	12-month	354	79	22.3	18.0–26.7
	18-month	468	74	15.8	12.5–19.1
Parechovirus	6-month	469	106	22.6	18.8–26.4
	12-month	354	60	17.0	13.0–20.9
	18-month	468	77	16.5	13.1–19.8
Rotavirus	6-month	469	15	3.2	1.6–4.8
	12-month	354	4	1.1	0.02–2.2
	18-month	468	3	0.6	−0.08 to 1.4
*Giardia lamblia*	6-month	469	45	9.6	6.9–12.3
	12-month	354	83	23.5	19.0–27.9
	18-month	468	123	26.3	22.3–30.3
*Cryptosporidium*	6-month	469	30	6.4	4.2–8.6
	12-month	354	28	7.9	5.1–10.7
	18-month	468	9	1.9	0.7–3.2

CI, confidence interval.

We also used Ct values to classify infections as low (Ct ⩾ 36), median (30 ⩽ Ct ⩽ 36) and high (Ct < 30) levels. The prevalence of microbes is highest in high infection levels in most of the viruses except rhinovirus in which the low infection level is the highest (data not shown).

### Predictors of viral and parasitic infections at 6-mon infants

In bivariate analyses, enterovirus positivity in the stool samples was significantly associated with sanitation (no household latrine use), site (living in Lungwena), lower level of mother's and father's education and rainy season (Table 3). In multivariate analysis, seasonality and father's education level remained significantly associated with enterovirus positivity (Table 3). Infants whose father had a higher level of education were less likely enterovirus positive than those whose father had a lower level of education (OR 0.94, 95% CI 0.89–0.99, *P* = 0.031). Infants whose sample was collected during the dry season (May–October) were less likely to be enterovirus positive than those whose sample was collected during the rainy season (November–April) (OR 0.65, 95% CI 0.43–0.99, *P* = 0.045) (Table 3). Age was associated with rhinovirus infections in multivariate analysis. Older infants were less likely rhinovirus positive than those young infants (OR 0.43, 95% CI 0.19–0.99, *P* = 0.046) (Table 3).

**Table 3.** Bivariate and multivariate analysis of potential predictors for enterovirus and rhinovirus among the participants

	Enterovirus bivariate		Enterovirus multivariate		Rhinovirus bivariate		Rhinovirus multivariate	
Variable	OR (95% CI)	*P* value	OR (95% CI)	*P* value	OR (95% CI)	*P* value	OR (95% CI)	*P* value
Age	0.50 (0.23–1.10)	0.084	0.64 (0.27–1.53)	0.32	0.46 (0.20–1.03)	0.06	0.43 (0.19–0.99)	0.046
Gender (girls)	1.15 (0.78–1.69)	0.491	1.19 (0.79–1.79)	0.418	1.11 (0.74–1.67)	0.6	1.16 (0.77–1.76)	0.485
Number of economic support person in the family	1.02 (0.84–1.23)	0.849	1.09 (0.90–1.33)	0.374	1.05 (0.87–1.27)	0.612	1.06 (0.87–1.28)	0.573
Sanitation (no household latrine)	2.72 (1.03–7.20)	0.044	2.40 (0.87–6.59)	0.089	0.68 (0.29–1.62)	0.385		
Site (Lungwena)	1.56 (1.05–2.32)	0.028	1.18 (0.75–1.87)	0.467	1.16 (0.76–1.77)	0.49		
Number of children < 5 years of age in household	1.28 (0.96–1.71)	0.096	1.19 (0.88–1.59)	0.258	1.12 (0.85–1.47)	0.435		
High maternal education level	0.91 (0.86–0.97)	0.002	0.96 (0.89–1.02)	0.208	0.99 (0.93–1.05)	0.665		
High parental education level	0.92 (0.87–0.97)	0.001	0.94 (0.89–0.99)	0.031	0.99 (0.94–1.04)	0.664		
Season (May–October)	0.64 (0.43–0.95)	0.027	0.65 (0.43–0.99)	0.045	1.04 (0.69–1.57)	0.843		
Number of animals in house	0.89 (0.75–1.07)	0.209	1.00 (0.98–1.02)	0.791	0.99 (0.97–1.01)	0.404	0.99 (0.97–1.01)	0.403

CI, confidence interval; OR, odds ratio.

Bivariate analysis also included WAZ, LAZ, WLZ, number of persons in household, anaemia, malaria, source of drinking water and age of main guardian (*N* = 455–469).

The results of norovirus, parechovirus and rotavirus are shown in Table 4. In bivariate analysis, the number of persons in the household, mother's education level, father's education level, anaemia and rainy season were significantly associated with norovirus presence. Anaemia was associated with parechovirus infections and lower level of sanitation (household latrine use) was associated with rotavirus infections (Table 4). In multivariate analysis, number of persons in the household and seasonality remained significantly associated with norovirus presence; and sanitation with rotavirus presence. The more person living in the house, the more likely infants were to have norovirus infection (OR 1.16, 95% CI 1.02–1.31, *P* = 0.024). Infants whose sample was collected during the dry season (May–October) were less likely norovirus positive than those whose sample was collected during the rainy season (November–April) (OR 0.53, 95% CI 0.32–0.86, *P* = 0.01). Infants whose home did not have latrine had four times higher odds of rotavirus being detected in their stool than those whose home had latrine (OR 4.11, 95% CI 1.13–14.88, *P* = 0.032) (Table 4).

**Table 4.** Bivariate and multivariate analysis of potential predictors for norovirus, parechovirus and rotavirus among the participants

	Norovirus bivariate		Norovirus multivariate		Parechovirus bivariate		Parechovirus multivariate		Rotavirus bivariate		Rotavirus multivariate	
Variable	OR (95% CI)	*P* value	OR (95% CI)	*P* value	OR (95% CI)	*P* value	OR (95% CI)	*P* value	OR (95% CI)	*P* value	OR (95% CI)	*P* value
Age	1.29 (0.55–3.02)	0.56	1.40 (0.58–3.34)	0.455	1.38 (0.58–3.28)	0.463	1.44 (0.61–3.44)	0.407	0.57 (0.07–4.38)	0.588	0.82 (0.10–6.97)	0.859
Gender (girls)	1.16 (0.75–1.78)	0.503	1.08 (0.68–1.71)	0.753	1.06 (0.69–1.63)	0.795	1.03 (0.65–1.62)	0.902	1.03 (0.37–2.88)	0.958	1.28 (0.43–3.79)	0.652
Number of economic support person in the family	0.98 (0.80–1.21)	0.87	1.07 (0.86–1.34)	0.552	1.05 (0.85–1.28)	0.669	1.10 (0.89–1.35)	0.384	0.93 (0.55–1.57)	0.79	1.00 (0.59–1.71)	0.992
Sanitation (no household latrine)	0.88 (0.37–2.10)	0.778			1.31 (0.59–2.92)	0.503			5.23 (1.57–17.44)	0.007	4.11 (1.13–14.88)	0.032
High maternal education level	0.92 (0.86–0.99)	0.017	0.95 (0.87–1.03)	0.183	1.02 (0.95–1.09)	0.579			0.88 (0.73–1.05)	0.16		
High parental education level	0.93 (0.88–0.99)	0.022	0.94 (0.88–1.01)	0.094	1.01 (0.96–1.07)	0.611			0.85 (0.72–1.01)	0.059	0.88 (0.74–1.04)	0.128
Season (May-October)	0.55 (0.35–0.87)	0.01	0.53 (0.32–0.86)	0.01	0.66 (0.42–1.04)	0.072	0.71 (0.44–1.13)	0.149	1.63 (0.58–4.58)	0.352		
Number of animals in house	0.98 (0.96–1.01)	0.199	0.98 (0.95–1.01)	0.169	0.99 (0.97–1.02)	0.512	0.99 (0.97–1.02)	0.461	0.95 (0.86–1.05)	0.307	0.96 (0.87–1.06)	0.44
Number of persons in household	1.13 (1.01–1.27)	0.032	1.16 (1.02–1.31)	0.024	1.06 (0.94–1.19)	0.322			0.92 (0.68–1.25)	0.6		
Anaemia	0.45 (0.22–0.95)	0.035	0.45 (0.20–1.01)	0.052	0.47 (0.23–0.99)	0.046	0.52 (0.24–1.10)	0.086	1.57 (0.43–5.76)	0.5		

CI, confidence interval; OR, odds ratio.

Bivariate analysis also included WAZ, LAZ, WLZ, site, number of children <5 years of age in household, age of main guardian and source of drinking water (*N* = 455–469).

For *Giardia lamblia* infection, the study site and mother's education level were associated with it in bivariate analysis (*P* = 0.015 and *P* = 0.012). In multivariate analysis, both associations were disappeared (*P* = 0.097 and *P* = 0.082) (Table 5).

**Table 5.** Bivariate and multivariate analysis of potential predictors for *Giardia lamblia* and *Cryptosporidium* among the participants

	*Giardia* bivariate		*Giardia* multivariate		*Cryptosporidium* bivariate		*Cryptosporidium* multivariate	
Variable	OR (95% CI)	*P* value	OR (95% CI)	*P* value	OR (95% CI)	*P* value	OR (95% CI)	*P* value
Age	0.91 (0.27–3.10)	0.882	1.19 (0.35–4.03)	0.781	0.82 (0.19–3.59)	0.795	1.78 (0.43–7.37)	0.424
Gender (girls)	0.93 (0.50–1.73)	0.826	1.01 (0.53–1.89)	0.987	2.20 (0.99–4.91)	0.054	2.86 (1.19–6.88)	0.019
Number of economic support person in the family	0.78 (0.55–1.11)	0.172	0.87 (0.60–1.25)	0.439	1.03 (0.73–1.45)	0.877	1.16 (0.80–1.69)	0.433
Sanitation (no household latrine)	1.30 (0.44–3.89)	0.636			0.43 (0.06–3.26)	0.414		
Site (Lungwena)	2.55 (1.20–5.43)	0.015	1.97 (0.88–4.39)	0.097	2.48 (0.99–6.19)	0.052	2.59 (0.97–6.89)	0.057
Number of children <5 years of age in household	1.22 (0.83–1.80)	0.301			0.88 (0.52–1.51)	0.645		
High maternal education level	0.87 (0.78–0.97)	0.012	0.90 (0.81–1.01)	0.082	0.95 (0.84–1.07)	0.359		
High parental education level	0.94 (0.87–1.03)	0.185			0.97 (0.88–1.08)	0.619		
Season (May–October)	0.61 (0.31–1.17)	0.137			0.33 (0.13–0.83)	0.018	0.30 (0.11–0.81)	0.018
Number of animals in house	0.99 (0.95–1.02)	0.489	0.99 (0.95–1.03)	0.644	0.99 (0.94–1.03)	0.537	0.97 (0.91–1.04)	0.393
WLZ	0.96 (0.71–1.30)	0.801			0.68 (0.47–0.99)	0.043	0.58 (0.39–0.87)	0.008
Age of main guardian	1.01 (0.97–1.04)	0.665			1.03 (1.00–1.07)	0.08	1.04 (0.99–1.09)	0.051

CI, confidence interval; OR, odds ratio.

Bivariate analysis also included WAZ, LAZ, number of persons in household, anaemia, malaria, and source of drinking water (*N* = 455–469).

*Cryptosporidium* (spp.) infections were significantly associated with seasonality and WLZ in bivariate analysis. In multivariate analysis, they remained independently associated with *Cryptosporidium* infections together with gender (Table 5). Infants who had higher WLZ were less likely infected with *Cryptosporidium* than those with lower WLZ (OR 0.58, 95% CI 0.39–0.87, *P* = 0.008). Infants whose samples were collected during the dry season (May–October) were less likely to be infected with *Cryptosporidium* than those whose samples were collected during the rainy season (November–April) (OR 0.3, 95% CI 0.11–0.81, *P* = 0.018). Girls were more likely infected with *Cryptosporidium* than boys (OR 2.86, 95% CI 1.19–6.88, *P* = 0.019).

## Discussion

Our study investigated the prevalence of selected common viruses and parasites in a population of asymptomatic 6, 12 and 18-month-old Malawian children. The study also assessed the environmental predictors of the presence of viruses and parasites among 6-month infants. We found a high prevalence of viral and parasitic infections in these children. Several predictors were found to be associated with the infections including seasonality of sample collection, sanitation conditions and father's education level.

In this study, we used real-time PCR to detect viruses and parasites. Due to the sensitivity of these assays we could detect also low-grade infections which may be common in asymptomatic infants and children. Compared with traditional methods such as cell culture, antigen detection or microscopic examination of viruses or parasites, our results give more representative picture of the epidemiology of these infections including asymptomatic ones.

The prevalence of viruses and parasites in our study were much higher than those studies carried out in high-income countries, and more comparable with studies in low-income countries. The enterovirus prevalence in our study was much higher than those reported in Norwegian [28] and Finnish infants [13, 29], but more comparable with Mongolia infants [30]. The same trend was for the prevalence of rhinovirus in our study *vs.* Finnish infants [21, 29]; parechovirus *vs.* Finnish infants [13, 31] and Norwegian infants [32]; norovirus *vs.* German [33], Korean [34], Cameroonian [12] and South African children [35]. The prevalence of *Giardia lamblia* in our study was also higher than reported in Bangladeshi healthy infants [36], but quite comparable with Ugandan asymptomatic infants [37]. *Cryptosporidium* (spp.) prevalence was also higher than previously reported from Spanish [38] and French children [14], and Chinese [15] and Bangladeshi infants [36]. Our results were also comparable with those in MAL-ED and GEMS non-diarrhoeal samples in norovirus [16, 18] and *Giardia* infections [17], but with a lower rate of rotavirus and *Cryptosporidium* infections in our study [18].

The discrepancy between our findings and those of other studies can to some extent be explained by the use of different methods for detecting the infections. In addition, other factors such as differences in study populations, personal and community hygienic standards, sanitation, climate, home crowding, water and food supplies, socioeconomic condition, seasonal variations, proximity to both domestic and wild animals and a high prevalence of other diseases and conditions such as HIV infection and malnutrition may have influenced the findings in different studies.

Only a few studies have investigated the predictors of viral and parasitic infections in healthy infants living in rural low-income settings. Our results are, however, in agreement with previous studies showing seasonality of enterovirus and *Cryptosporidium* infections [39–41]. There is also some previous evidence from Accra that the use of non-flush toilets is associated with a higher enteroviral isolation rate among healthy infants [39]. The absence of latrines has been associated with diarrhoea incidence also in Vietnam, where rotavirus was the most common causative agent [42]. These findings are consistent with the fecal-oral transmission route for these microbes and suggest that improvements in sanitary conditions might decrease the prevalence of enteric viral infections in rural Malawi.

Our study shows the high asymptomatic infections in Malawian children. These children carrying the pathogens did not have clinical symptoms and looked ‘healthy’ from their caretakers’ views when the stool samples were collected. It is possible that the high prevalence of the asymptomatic infections in these children is due to the frequent exposure to the pathogens which are common in low-income settings. In regards to *Cryptosporidium* and *Giardia lamblia*, some species does not infect human and possibly play a role in the high prevalence of parasitic asymptomatic infections in our study. To reduce the infection rate based on our findings, hygiene, better sanitation especially in the rainy season are important.

Taken together, our findings reveal a high prevalence of viruses and parasites in asymptomatic Malawian children. The high prevalence of infection could affect immune response and production of pro-inflammatory cytokines, nutritional status and the development of EED. Chronic infections may be asymptomatic in these children. However, the cumulative effect of these infections could be substantial. Future studies are needed to investigate if such infections and specific species of pathogens are associated with the development of EED and eventually lead to growth failure among infants and children in low-income settings.

## Supplementary material

For supplementary material accompanying this paper visit https://doi.org/10.1017/S0950268819000025. click here to view supplementary material
