# Supplementary material for: High prevalence of selected viruses and parasites and their predictors in Malawian children
Source: Epidemiol Infect. 2019 Feb 22;147:e90. doi: 10.1017/S0950268819000025 (PMC6521582; doi:10.1017/S0950268819000025)
Supplement: Supplementary file 1 [file S0950268819000025sup001.docx]

**Supplementary Table 1. PCR-primers and probes**

| **Virus/Parasites** | **Primer name** | **Sequence Concentration (µM)** | |
| --- | --- | --- | --- |
| **Enterovirus** | fwd 636 | CGGCCCCTGAATGCGGCTAA | **900** |
|  | rev 4- | GAAACACGGACACCCAAAGTA | **900** |
| **Rhinovirus** | HRVfwd | CYA* GCC T*GC GTG GC | **900** |
|  | HRVrv | GAA ACA CGG ACA CCC AAA GTA | **900** |
| **Norovirus** | NoroG2 fwdQ | CARGARBCNATGTTYAGRTGGATGAG | **900** |
|  | NoroG2 revQ | TCGACGCCATCTTCATTCACA | **300** |
|  | NoroG1 revQ: | CTTAGACGCCATCATCATTYAC | **900** |
|  | NoroG1 fwdQ | CGYTGGATGCGNTTYCATGA | **900** |
| **Rotavirus** | VP2-F1 | TCTGCAGACAGTTGAACCTATTAA | **900** |
|  | VP2-F2 | CAGACACGGTTGAACCCATTAA | **900** |
|  | VP2-F3 | TCGGCTGATACAGTAGAACCTATAAATG | **900** |
|  | VP2-F4 | TGTCAGCTGATACAGTAGAACCTATAAATG | **900** |
|  | VP2-F5 | TCAGCTGACACAGTAGAACCTATA AATG | **900** |
|  | VP2-R1 | GTTGGCGTTTACAGTTCGTTCAT | **50** |
|  | VP2-R2 | GTTGGCGTCTACAATTCGTTCAT | **50** |
| **Parechovirus** | ParE AN345 | GTAACASWWGCCTCTGGGSCCAAAAG | **300** |
|  | ParE AN344 | GGCCCCWGRTCAGATCCAYAGT | **300** |
| ***Giardia lamblia*** | FW | GAC GGC TCA GGA CAA CGG TT | **700 nM** |
|  | Rev | TTG CCA GCG GTG TCC G | **700 nM** |
| ***Cryptosporidium spp.*** | FW | CTT TTT ACC AAT CAC AGA ATC ATC AGA | **400 nM** |
|  | Rev | TGT GTT TGC CAA TGC ATA TGA A | **400 nM** |
| **Virus/Parasites** | **Probe** | **Sequence and label** |  |
| **Enterovirus** | Q-PCREVI | FAM-TCTGTGGCGGAACCGACTA-TAMRA | **300** |
|  | Q-PCREVII | FAM-TCTGCAGCGGAACCGACTA-TAMRA | **300** |
| **Rhinovirus** | Rhinoprobe | VIC-TCC TCC GGC CCC TGA ATG YGG C –TAMRA. | **300** |
| **Norovirus** | NoroG2probeQ | FAM-TGG GAG GGC GAT CGC AAT CT-TAMRA | **300** |
|  | NoroG1probeQ | VIC-AGA TYG CGA TCY CCT GTC CA- TAMRA | **250** |
| **Rotavirus** | RotaVp2-P | FAM-ATG CGC ATR TTR TCA AAH GCA A-MGB-NFQ | **200** |
| **Parechovirus** | ParE AN257 | FAM-CCTRYGGGTACCTYCWGGGCATCCTTC-TAMRA | **200** |
| ***Giardia lamblia*** |  | FAM-CCC GCG GCG GTC CCT GCT AG-MGB | **100 nM** |
| ***Cryptosporidium spp.*** |  | VIC-TCG ACT GGT ATC CCT ATA A-MGB | **100 nM** |

A* and T* locked nucleic acid primer by Exiqon
